# Investigation of *leptin receptor* rs1137101 G>A polymorphism with cancer risk: evidence from 35936 subjects

**DOI:** 10.1042/BSR20182240

**Published:** 2019-06-28

**Authors:** Guoxiang Rong, Weifeng Tang, Yafeng Wang, Hao Qiu, Shuchen Chen

**Affiliations:** 1 Department of Thoracic Surgery, The People’s Hospital of Danyang, Danyang, Jiangsu Province, China; 2 Department of Cardiothoracic Surgery, Affiliated People’s Hospital of Jiangsu University, Zhenjiang, Jiangsu Province, China; 3 Department of Cardiology, The People’s Hospital of Xishuangbanna Dai Autonomous Prefecture, Jinghong, Yunnan Province, China; 4 Institute of Laboratory Medicine, Jiangsu University, Zhenjiang, Jiangsu Province, China; 5 Department of Thoracic Surgery, Fujian Medical University Union Hospital, Fuzhou, Fujian Province, China

**Keywords:** cancer, energy, leptin, polymorphism, receptor, risk

## Abstract

Leptin receptor (LEPR) signaling may be involved in promoting angiogenesis and proliferation, inhibiting apoptosis and playing a vital role in the progression of carcinogenesis. A number of studies have focused on the association of *LEPR* rs1137101 variants with susceptibility of cancer, however, the observed results were controversial. We searched literature on the relationship of *LEPR* rs1137101 G>A polymorphism with cancer risk by using PubMed and Embase databases, covering all publications up to 14 October 2018. In total, 44 case–control studies with 35,936 subjects were included. After combining all eligible studies, we identified null relationship between *LEPR* gene rs1137101 G>A polymorphism and overall cancer risk [A *vs.* G: odds ratio (OR ) =  0.97, 95% confidence interval (CI ) =  0.89–1.06, *P* = 0.547; AA *vs.* GG: OR  =  0.93, 95% CI  =  0.78–1.13, *P* = 0.476; AA/GA *vs.* GG: OR  =  0.99, 95% CI  =  0.91–1.09, *P*= 0.890 and AA *vs.* GA/GG: OR  = 0.92, 95% CI  =  0.82–1.04, *P*= 0.198]. However, in a subgroup analysis, there was an increased susceptibility of oral and oropharyngeal cancer in AA *vs.* GA/GG genetic model (OR, 1.83; 95% CI, 1.01–3.33; *P*=0.048). Considering the limited participants were included, the findings might be underpowered. Sensitivity analysis identified that any independent study omitted did not materially influence the pooled ORs and CIs. The results of publication bias detection showed that there was no evidence of bias. In summary, this analysis indicates that no significant association of cancer risk was identified to be correlated with rs1137101 G>A variants, even in stratified analyses.

## Introduction

Cancer is one of the common health burden worldwide. Due to the prevalence of smoking and drinking, environmental pollution, as well as population aging, the incidence of malignancy is rising. According to the estimation of Global Cancer Statistics 2018, approximately 18.1 million new cancer cases and 9.6 million cancer-related deaths may have occurred in 2018 [1]. In the developing countries, the prognosis of cancer could be poorer than that in the developed countries. The reason of this phenomenon may be due to the diagnosis at an advanced stage combined with limited treatment. Some effective measures can potentially contribute to relieve the global cancer burden, including the application of precise early detection and treatment, tobacco and alcohol control, vaccine injection, sufficient fruits and vegetables intake and appropriate physical exercise. Overweight/obesity influences the health of more than 200 million people [2]. Overweight/obesity may play a vital role in growing morbidity of malignancy [3,4,5]. Thus, it is believed that overweight/obesity-related genes could affect the development of cancer.

Overweight/obesity is attributable to a chronic energy intake and expenditure imbalance. Leptin (LEP) is a common hormone of regulating energy expenditure by inhibiting hunger. LEP receptor (LEPR) is a type I cytokine receptor which is encoded by the *LEPR* gene and acts as a receptor for the hormone LEP. LEPR is a single transmembrane-domain receptor and composed of extracellular, transmembrane, and intracellular sections. LEP/LEPR signaling may involve in promoting angiogenesis, facilitating cell proliferation, and inhibiting epithelial cell apoptosis [6]. The long isoform in LEPR cytoplasmic domain may be essential for the signal transduction of Janus kinase/signal transducers and activators of transcription pathway [7].

The *LEPR* gene lies in chromosome 1 (Position_38: 65420652 – 65637493). There are a number of common single nucleotide polymorphisms (SNPs) of the *LEPR* genes, which have been established. *LEPR* rs1137101 G>A polymorphism (Arg223Gln) is the most extensively studied association of this SNP with the development of cancer. *LEPR* rs1137101 locus is a missense variant, which is a substitution of G→A at nucleotide number 668 in exon 6 of *LEPR* gene [8]. It leads to an Arg→Gln altering in extracellular region [8]. The potential relationships of *LEPR* rs1137101 variants with susceptibility of cancer have been elucidated in different malignancies; however, the obtained results were inconsistent. Two previous meta**-**analyses indicated that *LEPR* rs1137101 G>A polymorphism might not be a risk factor for cancer [9,10]. Recently, more studies concerning the association of *LEPR* rs1137101 locus with cancer risk were performed [11–26]. Hence, we carried out an updated meta-analysis on such relationship so as to further explore the role of *LEPR* rs1137101 variants to susceptibility of cancer.

## Materials and methods

### Searching publications

To obtain the potentially eligible investigations on *LEPR* rs1137101 G>A polymorphism and cancer susceptibility, we conducted an electronic literature search on PubMed and Embase databases, covering all publications up to 14 October 2018, by using the following searching strategy: (Leptin receptor or LEPR or obese receptor or OBR or CD295) and (carcinoma or cancer or tumor or malignancy or neoplasms) and (polymorphism or SNP or variation). References of the eligible studies and reviews were also screened to identify the additional data. We reported the present study using the Preferred Reporting Items for PRISMA guideline (Supplementary Table S1; PRISMA checklist) [27].

### Selection and exclusion criteria

The major selection criteria were: (i) full-text study, (ii) assessing the relationship of *LEPR* rs1137101 variants with cancer susceptibility, (iii) designed as an unrelated case–control study, (iv) sufficient data could be obtained to calculate the odds ratio (OR) with 95% confidence interval (CI), and (v) genotype distribution conformation to Hardy–Weinberg equilibrium (HWE).

The major exclusion criteria were: (i) genotype data could not be extracted; (ii) not case–control study; (iii) distribution of genotype violated HWE; and (iv) comments, reviews, and letters.

### Data extraction

Two authors (G.R. and Y.W.) independently extracted raw data. The following data were collected: the surname of first author, publication year, race, country, number of participants, age, Body Mass Index (BMI), source of control, matching method, genotype frequencies and genotyping method. Ethnicity descents were defined as mixed, Asian, and Caucasian. Cancer types were classified as hepatocellular carcinoma, breast cancer, renal cell carcinoma, esophageal cancer, colorectal cancer, oral and oropharyngeal cancer, non-Hodgkin’s lymphoma and other cancers (lung cancer and bladder cancer). For source of control, the eligible studies were categorized as hospital-based and population-based. When HWE in control group was not available, an online software (http://ihg.gsf.de/cgi-bin/hw/hwa1.pl) was harnessed to calculate the *P-*value of HWE test. If the information extracted was different, two authors reached consensus on each item.

### Statistical analysis

The relationship strength of *LEPR* rs1137101 SNP with susceptibility of cancer was determined by crude ORs with their 95% CIs. The pooled ORs were calculated for allele (A vs. G), dominant (GA/AA vs. GG), recessive (AA versus GA/GG), and homozygote comparison (AA vs. GG) genetic modes. Additionally, stratified analysis was carried out to assess the influence of confounding risk factors: ethnicity (mixed, Asian, and Caucasian) and cancer type (breast cancer, esophageal cancer, hepatocellular carcinoma, renal cell carcinoma, colorectal cancer, oral and oropharyngeal cancer, non-Hodgkin’s lymphoma, and other cancers). We used Chi-square based Q-test and *I^2^* test to assess the potential heterogeneity among the eligible studies. *P*<0.10 or *I^2^* ≥ 50% was considered as the level of significant heterogeneity. Then a random-effects model (DerSimonian and Laird method) was used to assess the association of *LEPR* rs1137101 polymorphism with cancer susceptibility [28,29]; otherwise, a fixed-effects model (Mantel–Haenszel method) was harnessed to pool the data [30]. Stratified analysis was conducted to explore the source of heterogeneity. We performed one-way sensitivity analysis to evaluate the effect of an individual study on pooled ORs and CIs. Begg’s funnel plot and the Egger’s test were used to detect the potential bias in included publications, and a *P*<0.10 was considered significant [31,32]. Statistical analysis of the present study was calculated with STATA 12.0 software (StataCorp, College Station, Texas, U.S.A.).

## Results

### Study characteristics

**Figure 1:**
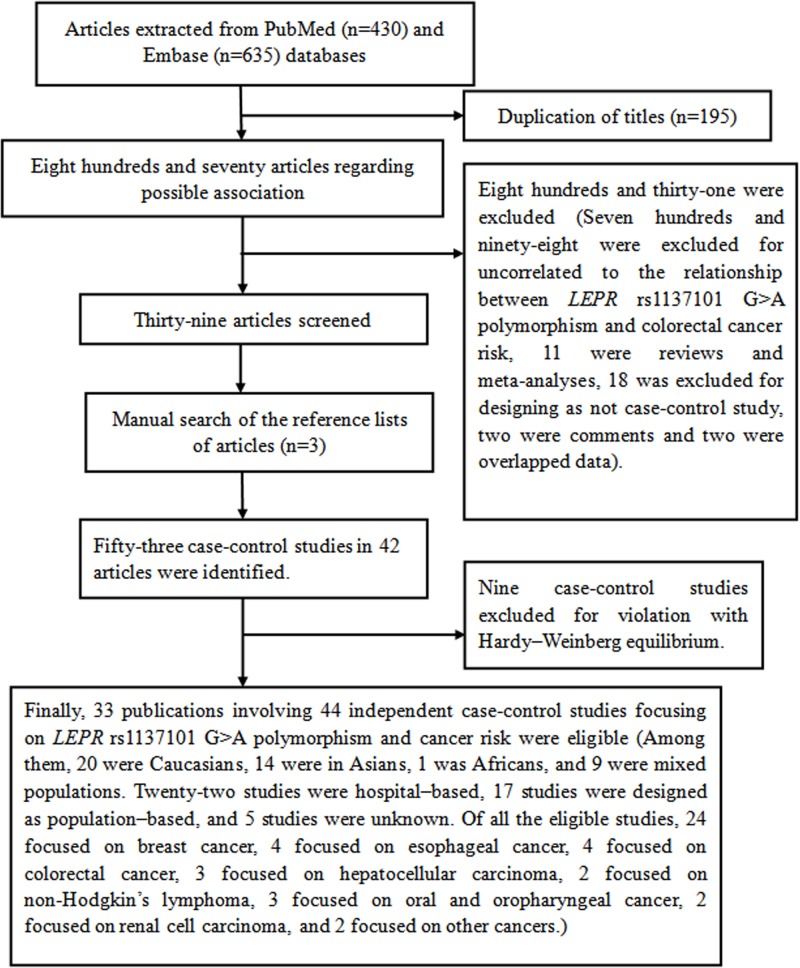
Flow diagram of the meta–analysis of the association between *LEPR* rs1137101 G>A polymorphism and cancer risk

In this meta-analysis, 33 publications involving 44 independent case–control studies on the relationship of *LEPR* rs1137101 G>A polymorphism with cancer risk were recruited [9,11–26,33–48]. In some publications, they contained several subgroups that we treated as independent studies [12,14,40]. Figure 1 shows the eligible study selecting process. In total, 44 independent case–control studies with 35,936 subjects (13,711 cases and 22,225 controls) were included. Among them, 20 were Caucasians [15,16,20–23,34,36,37,39–43,45–48], 14 were Asians [9,11–14,17,24,26,33,44], 1 was African [38], and 9 were mixed populations [18,19,25,35]. Twenty-two case–control studies were designed as hospital-based investigation [9,11–14,17,18,21,22,24,26,33,34,38,44,46,48], seventeen studies were designed as population-based investigation [16,19,25,35–37,40,41,43,45,47], and the source of control in other five studies were unknown [15,20,23,39,42]. Of all the eligible studies, 24 focused on breast cancer [12,14,15,19,20,22,33,35,36,38,41,42,44,47,48], 4 focused on esophageal cancer [13,40], 4 focused on colorectal cancer [34,37,39,46], 3 focused on hepatocellular carcinoma [9,11,26], 2 focused on non-Hodgkin’s lymphoma [43,45], 3 focused on oral and oropharyngeal cancer [18,21,25], 2 focused on renal cell carcinoma [17,24], and 2 focused on other cancers [16,23]. The detailed information of eligible studies is listed in Table 1. The extracted genotypes and HWE are summarized in Table 2.

**Table 1:** Characteristics of the studies in meta-analysis

Study	Publication year	Country	Ethnicity	Cancer type	Sample size (case/control)	Case age (years)	Control age (years)	Case BMI (kg/m^2^)	Control BMI (kg/m^2^)	Source of control	Match	Genotype method
Zhang et al.	2018	China	Asians	Hepatocellular carcinoma	584/923	53.17 ± 11.76	53.72 ± 9.97	NA	NA	Hospital-based	Age, sex, ethnicity	SNPscan
Liu et al.	2018	China	Asians	Breast cancer	488/463	43.71 ± 6.13	43.35 ± 5.43	BMI < 24: *n*=300, BMI ≥ 24, *n*=148	BMI < 24: *n*=289, BMI ≥ 24, *n*=174	Hospital-based	Age, sex, ethnicity, region	MS-TOF
Liu et al.	2018	China	Asians	Breast cancer	346/342	58.55 ± 6.87	56.60 ± 6.53	BMI < 24:*n*=207, BMI ≥ 24, *n*=139	BMI < 24: *n*=195, BMI ≥ 24, *n*=147	Hospital-based	Sex, ethnicity, region	MS-TOF
Qiu et al.	2017	China	Asians	Esophageal cancer	507/1,496	62.77 ± 8.01	62.77 ± 8.84	22.27 ± 2.90	23.91 ± 3.03	Hospital-based	Age, ethnicity, sex	SNPscan
Yuna et al.	2018	China	Asians	Breast cancer	77/805	51.43 ± 11.33	48.98 ± 8.83	Obesity (≥27): *n*=15, non-obesity, *n*=62	Obesity (≥27), *n*=50, non-obesity, *n*=751	Hospital-based	Age, sex, ethnicity, region	MS-TOF
Yuna et al.	2019	China	Asians	Breast cancer	79/805	49.94 ± 10.10	48.98 ± 8.83	Obesity (≥27), *n*=12, non-obesity, *n*=67	Obesity (≥27), *n*=50, non-obesity, *n*=751	Hospital-based	Age, sex, ethnicity, region	MS-TOF
Yuna et al.	2017	China	Asians	Breast cancer	412/805	49.73 ± 9.38	48.98 ± 8.83	Obesity (≥27), *n*=45, non-obesity, *n*=365	Obesity (≥27), *n*=50, non-obesity, *n*=751	Hospital-based	Age, sex, ethnicity, region	MS-TOF
Yuna et al.	2017	China	Asians	Breast cancer	135/805	50.50 ± 9.04	48.98 ± 8.83	Obesity (≥27), *n*=12, non-obesity, *n*=123	Obesity (≥27), *n*=50, non-obesity, *n*=751	Hospital-based	Age, sex, ethnicity, region	MS-TOF
El-Hussiny et al.	2017	Egypt	Caucasians	Breast cancer	48/48	47.7 ± 7.5	43.5 ± 9.2	34.37 ± 6.08	27.28 ± 3.52	NA	Sex, ethnicity, region	PCR-RFLP
Ali et al.	2017	Pakistan	Caucasians	Bladder cancer	200/200	55.5 ± 13.24	54.3 ± 9.9	NA	NA	Population-based	Age, sex, ethnicity	PCR
Zhang et al.	2016	China	Asians	Renal cell carcinoma	83/161	17–85 (median: 57)	NA	NA	NA	Hospital-based	Ethnicity, age, and sex	PCR-RLFP
Rodrigues et al.	2015	Brazil	Mixed	Oral and oropharyngeal cancer	129/186	54.9 ± 10.7	54.2 ± 11.1	NA	NA	Hospital-based	Sex, region	PCR-RLFP
Slattery et al.	2015	America	Mixed	Breast cancer	239/252	NA	NA	BMI < 25	BMI < 25	Population-based	Sex, region	A multiplexed bead array assay
Slattery et al.	2015	America	Mixed	Breast cancer	176/150	NA	NA	BMI = 25–29	BMI = 25–29	Population-based	Sex, region	A multiplexed bead array assay
Slattery et al.	2015	America	Mixed	Breast cancer	111/126	NA	NA	BMI **≥ 30**	BMI **≥ 30**	Population-based	Sex, region	A multiplexed bead array assay
Slattery et al.	2015	America	Mixed	Breast cancer	253/239	NA	NA	BMI < 25	BMI < 25	Population-based	Sex, region	A multiplexed bead array assay
Slattery et al.	2015	America	Mixed	Breast cancer	205/304	NA	NA	BMI = 25–29	BMI = 25–29	Population-based	Sex, region	A multiplexed bead array assay
Slattery et al.	2015	America	Mixed	Breast cancer	148/224	NA	NA	BMI ≥ 30	BMI ≥ 30	Population-based	Sex, region	A multiplexed bead array assay
Mahmoudi et al.	2015	Iran	Caucasians	Breast cancer	45/41	47.09 ± 11.45	48.37 ± 8.80	NA	NA	NA	Age, sex	PCR-RFLP
Hussain et al.	2015	India	Caucasians	Oral carcinoma	306/228	33.5 ± 5.79	32.7 ± 5.73	29.5 ± 5.44	23.8 ± 4.88	Hospital-based	Age, sex, ethnicity and low-risk environment	PCR-RFLP
Mohammadzadeh et al.	2014	Iran	Caucasians	Breast cancer	100/100	48.16 ± 10.47	49.0 ± 7.77	27.16 ± 3.96	28.31 ± 4.71	Hospital-based	Age, sex, ethnicity, BMI	PCR-RFLP
Unsal et al.	2014	Turkey	Caucasians	Lung cancer	162/130	60.96 ± 11.88	57.92 ± 14.96	NA	NA	NA	Age, sex, ethnicity	PCR-RFLP
Mu et al.	2014	China	Asians	Renal cell carcinoma	77/161	56.22 ± 12.27	NA	NA	NA	Hospital-based	Ethnicity, age and sex	PCR-RFLP or DNA sequence
Domingos et al.	2014	Brazil	Mixed	Oral carcinoma	25/89	58.0 ± 13.6	55.9 ± 13.9	NA	NA	Population-based	Age, sex, and smoking habits	RFLP-PCR
Li et al.	2012	China	Asians	Hepatocellular carcinoma	417/551	52.45 ± 4.6	51.95 ± 2.8	21.44 ± 3.4	22.56 ± 3.2	Hospital-based	Age, sex, ethnicity	RFLP
Kim et al.	2012	Korea	Asians	Breast cancer	400/452	≤49: 64.8%	≤49: 62.4%	≤25: 68.5%	≤25: 78.5%	Hospital-based	Age, sex	MS-TOF
Karimi et al.	2011	Iran	Caucasians	Colorectal cancer	173/173	55.8 ± 12.7	44.8 ± 17.2	25.1 ± 5.3	26.2 ± 7.19	Hospital-based	BMI, sex and smoking status	RFLP
Nyante et al.	2011	America	Mixed	Breast cancer	1972/1776	23–74, mean: 50	21–74, mean: 51	BMI < 25, *n*=712; BMI ≥ 25, *n*=1219	BMI < 25, *n*=545; BMI ≥ 25, *n*=1194	Population-based	Age, sex and region	Illumina
Cleveland et al.	2010	America	Caucasians	Breast cancer	1065/1108	NA	NA	BMI < 30, *n*=291; BMI ≥ 30, *n*=43	BMI < 30, *n*=304; BMI ≥ 30, *n*=62	Population-based	Age, sex, region	PCR
Pechlivanis et al.	2009	Czech Republic	Caucasians	Colorectal cancer	702/752	27–85, mean: 62	29–91, mean: 54	13.1–44.9, mean: 26.5	16.6–44.3, mean: 26.4	Hospital-based	Sex, region	TaqMan
Okobia et al.	2008	Nigeria	Africans	Breast cancer	209/209	46.1 ± 12.63	47.1 ± 13.50	NA	NA	Hospital-based	Sex, region	PCR-RFLP
Vasků et al.	2009	Czech Republic	Caucasians	Colorectal cancer	102/101	68 ± 10.2	68.1 ± 5.4	Male/Female: 26.7 ± 5.1/26.9 ± 5.2	NA	NA	Age, ethnicity	PCR-RFLP
Doecke et al.	2008	Australia	Caucasians	Esophageal cancer	260/1352	NA	NA	NA	NA	Population-based	Sex, region	Sequenom
Doecke et al.	2008	Australia	Caucasians	Esophageal cancer	301/1352	NA	NA	NA	NA	Population-based	Sex, region	Sequenom
Doecke et al.	2008	Australian	Caucasians	Esophageal cancer	213/1352	NA	NA	NA	NA	Population-based	Sex, region	Sequenom
Gallicchio et al.	2007	America	Caucasians	Breast cancer	61/933	mean: 59	mean: 59	NA	BMI < 25, *n*=479; BMI ≥ 25, *n*=513	Population-based	NA	TaqMan
Snoussi et al.	2006	Tunisia	Caucasians	Breast cancer	308/222	50 ± 24	48 ± 14	NA	NA	NA	Sex, region	PCR-RFLP
Willett et al.	2005	U.K.	Caucasians	Non-Hodgkin’s lymphoma	1073/754	18–65	18–65	BMI < 25, *n*=633, BMI ≥ 25, *n*=603	BMI < 25, *n*=524; BMI ≥ 25, *n*=387	Population-based	Sex, region	TaqMan
Woo et al.	2006	Korea	Asians	Breast cancer	45/45	NA	NA	BMI < 25, *n*=27, BMI ≥ 25, *n*=18	BMI < 25, *n*=37; BMI ≥ 25, *n*=8	Hospital-based	Age, sex	DNA sequencing
Skibola et al.	2004	America	Caucasians	Non-Hodgkin’s lymphoma	376/805	21–74	NA	BMI < 25, *n*=213; BMI ≥ 25, *n*=162	BMI < 25: *n*=480; BMI ≥ 25, *n*=321	Population-based	Age, sex and region	TaqMan
Mahmoudi et al.	2016	Iran	Caucasians	Colorectal cancer	261/339	56.1 ± 12.6	44.3 ± 16.3	25.6 ± 4.9	25.2 ± 4.2	Hospital-based	Sex and BMI	PCR-RFLP
Dai et al.	2010	China	Asians	Hepatocellular carcinoma	80/102	32–65	28–60	NA	NA	Hospital-based	Age, sex, ethnicity	PCR-RFLP
Teras et al.	2009	America	Caucasians	Breast cancer	648/659	mean: 69	mean: 69	NA	NA	Population-based	Age, sex	SNPstream
Kuptsova et al.	2008	Russia	Caucasians	Breast cancer	110/105	56–65	50–70	NA	NA	Hospital-based	Age, sex	PCR

Abbreviations: MS-TOF, mass spectrometry time of flight; NA, not available; PCR, polymerase chain reaction; PCR-RFLP, PCR-restriction fragment length polymorphism.

**Table 2:** Distribution of *LEPR* rs1137101 G>A polymorphism genotype and allele

Study	Publication year	Case			Control			Case		Control		HWE
		AA	AG	GG	AA	AG	GG	A [*n* (%)]	G A [*n* (%)]	A A [*n* (%)]	G A [*n* (%)]	
Zhang et al.	2018	3	119	453	10	193	717	125 (10.87)	1025 (89.13)	213 (11.58)	1627 (88.42)	Yes
Liu et al.	2018	AA/AG = 101	NA	346	AA/AG = 99	NA	363	NA	NA	NA	NA	Yes
Liu et al.	2018	AA/AG = 80	NA	264	AA/AG = 79	NA	260	NA	NA	NA	NA	Yes
Qiu et al.	2017	6	108	390	21	322	1146	120 (11.90)	888 (88.10)	364 (12.22)	2,614 (87.78)	Yes
Yuan et al.	2017	AA/AG = 18	NA	59	AA/AG = 178	NA	623	NA	NA	NA	NA	Yes
Yuan et al.	2017	AA/AG = 17	NA	62	AA/AG = 178	NA	623	NA	NA	NA	NA	Yes
Yuan et al.	2017	AA/AG = 96	NA	314	AA/AG = 178	NA	623	NA	NA	NA	NA	Yes
Yuan et al.	2017	AA/AG = 30	NA	105	AA/AG = 178	NA	623	NA	NA	NA	NA	Yes
El-Hussiny et al.	2017	9	15	24	2	24	22	33 (34.38)	63, 65.63)	28 (29.17)	68 (70.83)	Yes
Ali et al.	2017	51	96	53	67	97	36	198 (49.50)	202 (50.50)	231 (57.75)	169 (42.25)	Yes
Zhang et al.	2016	2	21	61	7	52	102	25 (14.88)	143 (85.12)	66 (20.50)	256 (79.50)	Yes
Rodrigues et al.	2015	60	61	8	68	92	26	181 (70.16)	77 (29.84)	228 (61.29)	144 (38.71)	Yes
Slattery et al.	2015	77	NA	AG/GG:162	69	NA	AG/GG:183	NA	NA	NA	NA	Yes
Slattery et al.	2015	50	NA	AG/GG:126	50	NA	AG/GG:100	NA	NA	NA	NA	Yes
Slattery et al.	2015	22	NA	AG/GG:89	38	NA	AG/GG:88	NA	NA	NA	NA	Yes
Slattery et al.	2015	63	NA	AG/GG:190	80	NA	AG/GG:159	NA	NA	NA	NA	Yes
Slattery et al.	2015	46	NA	AG/GG:159	83	NA	AG/GG:221	NA	NA	NA	NA	Yes
Slattery et al.	2015	43	NA	AG/GG:105	52	NA	AG/GG:172	NA	NA	NA	NA	Yes
Mahmoudi et al.	2015	19	25	1	17	18	6	63 (70.00)	27 (30.00)	52 (63.41)	30 (36.59)	Yes
Hussain et al.	2015	48	110	148	12	72	144	206 (33.66)	406 (66.34)	96 (21.05)	360 (78.95)	Yes
Mohammadzadeh et al.	2014	25	56	19	54	40	6	106 (53.000)	94 (47.00)	148 (74.00)	52 (26.00)	Yes
Unsal et al.	2014	75	62	25	56	55	19	212 (65.43)	112 (34.57)	167 (64.23)	93 (35.77)	Yes
Mu et al.	2014	2	20	55	4	41	116	24 (15.58)	130 (84.42)	49 (15.22)	273 (84.78)	Yes
Domingos et al.	2014	12	7	6	40	38	11	31 (62.00)	19 (38.00)	118 (66.29)	60 (33.71)	Yes
Li et al.	2012	87	208	122	189	256	106	382 (45.80)	452 (54.20)	634 (57.53)	468 (42.47)	Yes
Kim et al.	2012	8	88	294	6	91	350	104 (13.33)	676 (86.67)	103 (11.52)	791 (88.48)	Yes
Karimi et al.	2011	77	75	21	67	80	26	229 (66.18)	117 (33.82)	214 (61.85)	132 (38.15)	Yes
Nyante et al.	2011	494	952	526	416	874	485	1940 (49.19)	2004 (50.86)	1706 (48.06)	1844 (51.94)	Yes
Cleveland et al.	2010	173	521	355	187	551	360	867 (41.33)	1231 (58.67)	925 (42.12)	1271 (57.88)	Yes
Pechlivanis et al.	2009	179	320	140	202	361	143	678 (53.05)	600 (46.95)	765 (54.18)	647 (45.82)	Yes
Okobia et al.	2008	46	107	56	56	107	46	199 (47.61)	219 (52.39)	219 (52.39)	199 (47.61)	Yes
Vasků et al.	2009	23	56	21	34	45	21	102 (51.00)	98 (49.00)	113 (56.50)	87 (43.50)	Yes
Doecke et al.	2008	73	140	47	419	663	270	286 (55.00)	234 (45.00)	1501 (55.51)	1203 (44.49)	Yes
Doecke et al.	2008	84	164	62	419	663	270	332 (53.55)	288 (46.45)	1501 (55.51)	1203 (44.49)	Yes
Doecke et al.	2008	64	106	43	419	663	270	234 (54.93)	192 (45.07)	1501 (55.51)	1203 (44.49)	Yes
Gallicchio et al.	2007	14	24	15	278	443	151	52 (49.07)	54 (50.94)	999 (57.28)	745 (42.72)	Yes
Snoussi et al.	2006	98	145	65	102	90	30	341 (55.36)	275 (44.64)	294 (66.22)	150 (33.78)	Yes
Willett et al.	2005	336	554	183	234	387	133	1226 (57.13)	920 (42.87)	855 (56.70)	653 (43.30)	Yes
Woo et al.	2006	0	12	33	0	8	37	12 (13.33)	78 (86.67)	8 (8.89)	82 (91.11)	Yes
Skibola et al.	2004	115	173	87	226	379	198	403 (53.73)	347 (46.27)	831 (51.74)	775 (48.26)	Yes
Mahmoudi et al.	2016	127	101	33	146	147	46	355 (68.01)	167 (31.99)	439 (64.75)	239 (35.25)	Yes
Dai et al.	2010	10	14	58	2	19	81	34 (20.73)	130 (79.27)	23 (11.27)	181 (88.73)	Yes
Teras et al.	2009	AA/AG = 460	NA	181	AA/AG = 439	NA	211	NA	NA	NA	NA	Yes
Kuptsova et al.	2008	17	69	24	18	51	36	103 (46.82)	117 (53.18)	87 (41.43)	123 (58.57)	Yes

Abbreviation: NA, not available.

### Results of meta-analysis

**Figure 2:**
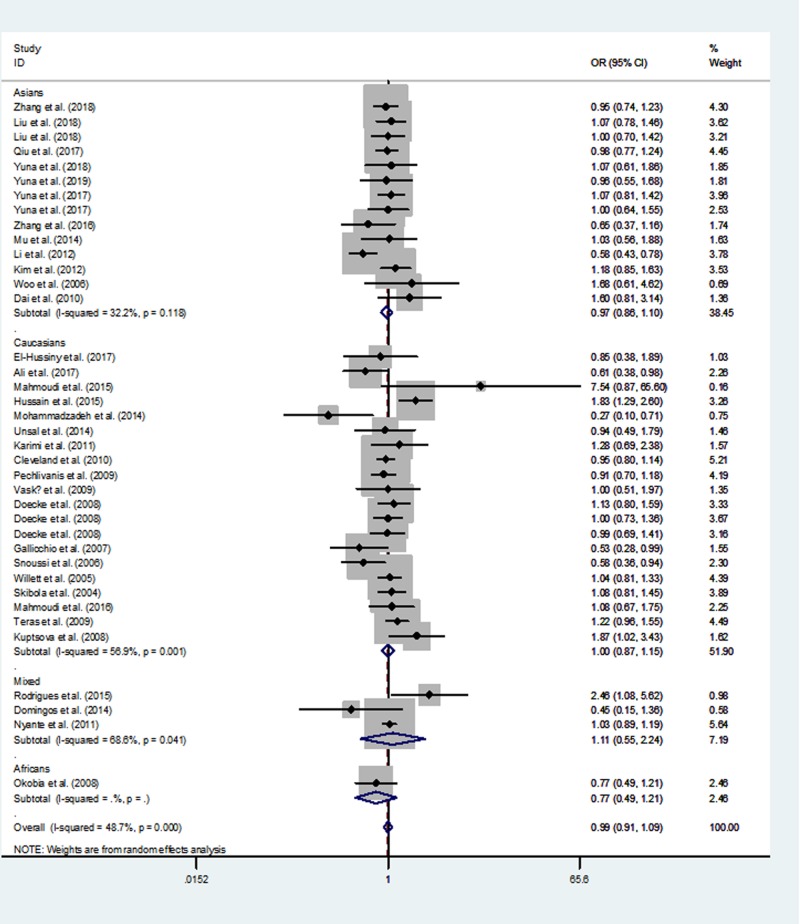
Meta-analysis of the association between *LEPR* rs1137101 G>A polymorphism and cancer risk (AA/GA vs. GG, random–effects model)

**Figure 3:**
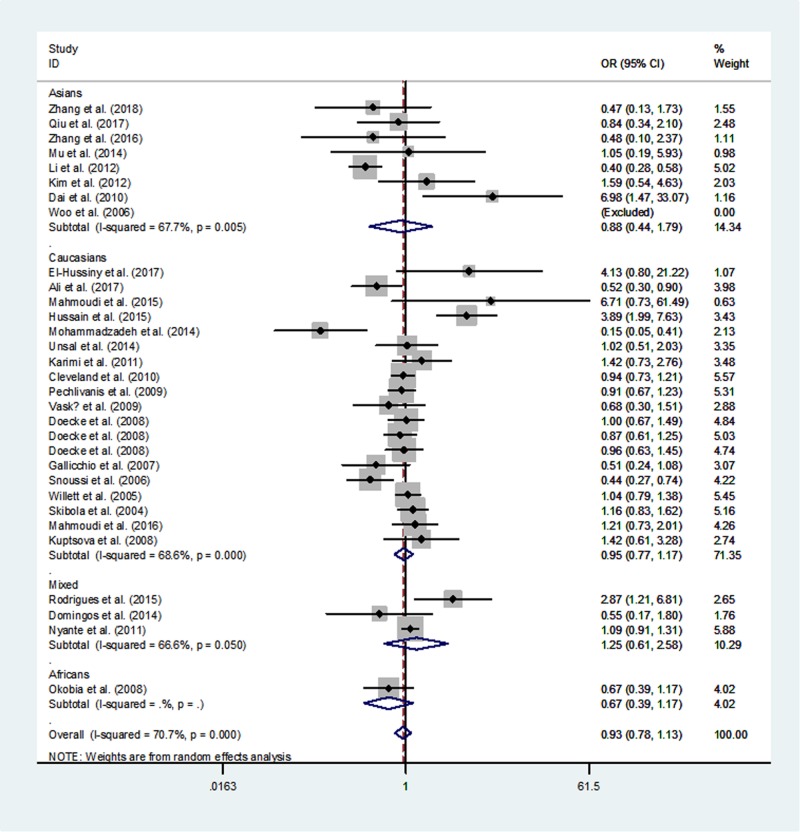
Meta-analysis of the association between *LEPR* rs1137101 G>A polymorphism and cancer risk (AA vs. GG, random–effects model)

**Figure 4:**
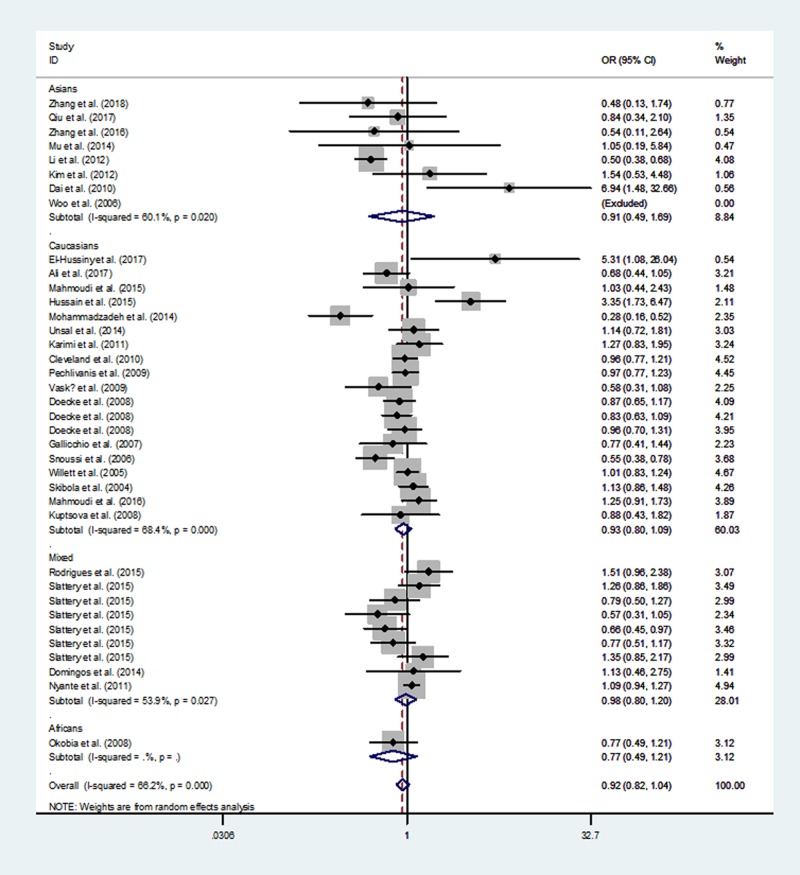
Meta-analysis of the association between *LEPR* rs1137101 G>A polymorphism and cancer risk (AA vs. GG/GA, random–effects model)

**Figure 5:**
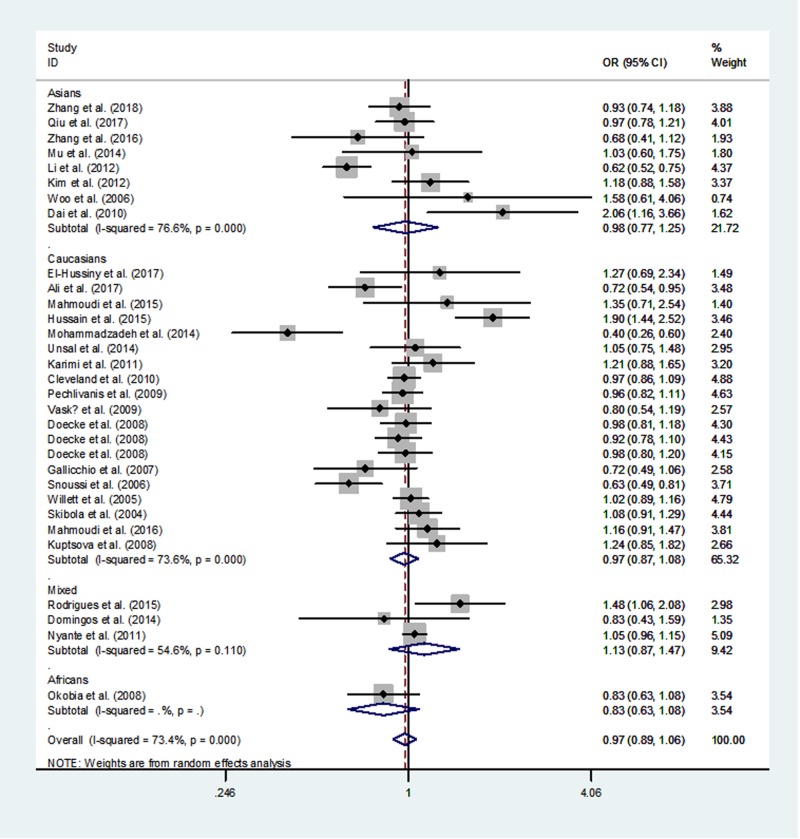
Meta-analysis of the association between *LEPR* rs1137101 G>A polymorphism and cancer risk (A vs. G, random–effects model)

Table 3 lists the overall and subgroup analysis results of the present study. After combining all eligible case–control studies, we identified null relationship between rs1137101 polymorphism in *LEPR* gene and overall cancer risk under four genetic models (A *vs.* G: OR  =  0.97, 95% CI  =  0.89–1.06, *P*=0.547; AA *vs.* GG: OR  =  0.93, 95% CI  =  0.78–1.13, *P* =0.476; AA/GA *vs.* GG: OR  =  0.99, 95% CI  =  0.91–1.09, *P*=0.890 and AA *vs.* GA/GG: OR  = 0.92, 95% CI  =  0.82–1.04, *P*=0.198, Figures 2–5).

**Table 3:** Results of the meta-analysis from different genetic models

	Number of studies	A vs. G				AA vs. GG				AA+GA vs. GG				AA vs.GA+GG			
		OR (95% CI)	*P*	*I^2^*	*P* (Q-test)	OR (95% CI)	*P*	*I^2^*	*P* (Q-test)	OR (95% CI)	*P*	*I^2^*	*P* (Q-test)	OR (95% CI)	*P*	*I^2^*	*P* (Q-test)
Total	44	0.97 (0.89–1.06)	0.547	73.4%	<0.001	0.93 (0.78–1.13)	0.476	70.7%	<0.001	0.99 (0.91–1.09)	0.890	48.7%	<0.001	0.92 (0.82–1.04)	0.198	66.2%	<0.001
**Ethnicity**																	
Caucasians	20	0.97 (0.87–1.08)	0.565	73.6%	<0.001	0.95 (0.77–1.17)	0.621	68.6%	<0.001	1.00 (0.87–1.15)	0.976	56.9%	0.001	0.93 (0.80–1.09)	0.381	68.4%	<0.001
Asians	14	0.98 (0.77–1.25)	0.864	76.6	<0.001	0.88 (0.44–1.79)	0.733	67.7%	0.005	0.96 (0.87–1.06)	0.456	32.2%	0.118	0.91 (0.49–1.69)	0.765	60.1%	0.020
Mixed	9	1.13 (0.87–1.47)	0.369	54.6%	0.110	1.25 (0.61–2.58)	0.539	60.6%	0.050	1.11 (0.55–2.24)	0.775	68.6%	0.041	0.98 (0.80–1.20)	0.837	53.9%	0.027
Africans	1	0.83 (0.63–1.08)	0.167	-	-	0.67 (0.39–1.17)	0.162	-	-	0.77 (0.49–1.21)	0.255	-	-	0.77 (0.49–1.21)	0.255	-	-
**Cancer type**																	
Breast cancer	24	0.91 (0.77–1.07)	0.248	75.5%	<0.001	0.82 (0.57–1.17)	0.269	74.6%	<0.001	1.00 (0.88–1.13)	0.946	44.1%	0.024	0.84 (0.69–1.02)	0.076	67.3%	<0.001
Colorectal cancer	4	1.01 (0.90–1.14)	0.814	30.4%	0.230	1.00 (0.79–1.26)	0.967	0.0%	0.398	0.98 (0.80–1.21)	0.860	0.0%	0.742	1.04 (0.88–1.23)	0.623	48.2%	0.122
Esophageal cancer	4	0.96 (0.87–1.06)	0.407	0.0%	0.967	0.93 (0.75–1.16)	0.525	0.0%	0.957	1.01 (0.87–1.18)	0.873	0.0%	0.916	0.88 (0.74–1.03)	0.118	0.0%	0.925
Hepatocellular carcinoma	3	0.98 (0.60–1.62)	0.948	89.4%	<0.001	0.96 (0.21–4.42)	0.956	84.1%	0.002	0.89 (0.55–1.44)	0.633	80.8%	0.005	1.03 (0.25–4.14)	0.971	81.5%	0.005
Oral and oropharyngeal cancer	3	**1.46 (1.00–2.13)**	**0.050**	64.1%	0.062	2.02 (0.72–5.65)	0.179	75.1%	0.018	1.43 (0.67–3.07)	0.360	69.1%	0.039	**1.83 (1.01–3.33)**	**0.048**	60.6%	0.079
Renal cell carcinoma	2	0.82 (0.57–1.18)	0.291	19.7%	0.264	0.67 (0.21–2.14)	0.500	0.0%	0.509	0.81 (0.53–1.23)	0.318	13.9%	0.281	0.72 (0.22–2.28)	0.570	0.0%	0.576
Non-Hodgkin’s lymphoma	2	1.04 (0.94–1.16)	0.451	0.0%	0.577	1.09 (0.88–1.35)	0.438	0.0%	0.641	1.06 (0.88–1.28)	0.547	0.0%	0.839	1.05 (0.90–1.24)	0.528	0.0%	0.526
Others	2	0.86 (0.59–1.25)	0.426	65.9%	0.087	0.68 (0.44–1.04)	0.076	55.3%	0.135	0.71 (0.48–1.04)	0.079	9.8%	0.292	0.86 (0.63–1.18)	0.600	60.7%	0.111
**Sample size**																	
<1000	32	0.98 (0.82–1.17)	0.816	81.1%	<0.001	0.98 (0.67–1.43)	0.913	78.6%	<0.001	0.97 (0.81–1.15)	0.694	62.4%	<0.001	0.93 (0.75–1.14)	0.466	72.9%	<0.001
≥1000	12	1.00 (0.95–1.05)	0.950	0.0%	0.926	1.01 (0.91–1.11)	0.906	0.0%	0.882	1.02 (0.95–1.09)	0.548	0.0%	0.945	1.00 (0.92–1.08)	0.907	0.0%	0.695
**Source of control**																	
Hospital-based	22	1.03 (0.86–1.23)	0.744	82.7%	<0.001	1.04 (0.68–1.57)	0.869	80.2%	<0.001	1.03 (0.90–1.19)	0.651	57.4%	<0.001	0.99 (0.72–1.36)	0.965	78.6%	<0.001
Population-based	17	0.99 (0.94–1.04)	0.715	20.9%	0.251	0.99 (0.87–1.03)	0.787	25.0%	0.214	1.01 (0.94–1.10)	0.754	29.3%	0.167	0.97 (0.90–1.04)	0.368	29.1%	0.132
NA	5	0.92 (0.68–1.23)	0.560	61.7%	0.034	1.02 (0.48–2.13)	0.968	68.6%	0.013	0.82 (0.61–1.10)	0.190	38.7%	0.163	0.89 (0.53–1.48)	0.649	69.4%	0.011

*I^2^*: ≥50% indicate significant heterogeneity. *P* (Q-test): <0.10 considered as the criterion of significant heterogeneity.

Abbreviation: NA, not available. Bold values are statistically significant (*P*<0.05).

When we conducted a stratified analysis by ethnicity, null association was found in mixed populations, Africans, Asians, and Caucasians. However, when we performed a subgroup analysis by cancer type, there was an increased susceptibility of oral and oropharyngeal cancer in AA *vs.* GA/GG genetic model (OR, 1.83; 95% CI, 1.01–3.33; *P*=0.048).

### Heterogeneity analysis

Significant heterogeneity among the eligible studies was found in this pooled analysis (Table 3). In the present study, we carried out subgroup analysis to explore the sources of heterogeneity. We found that Caucasians, breast cancer, hepatocellular carcinoma, other cancers, small sample sizes (<1000) and hospital-based case–control studies might contribute the major source to heterogeneity.

### Sensitivity analysis

Sensitivity analysis of one-way method identified that any individual study deleted did not materially influence the pooled ORs and CIs under all genetic comparisons. These findings indicated that our observations were stable and reliable (Supplementary Figure S1).

### Publication bias

Begg’s funnel plot and Egger’s test were used to evaluate the potential bias. The results of the bias detecting were shown as following: A *vs.* G: *P* _Begg’s_= 0.852, *P*_Egger’s_ = 0.973; AA *vs.* GG: *P* _Begg’s_= 0.775, *P*_Egger’s_ = 0.897; AA/GA *vs.* GG: *P* _Begg’s_ = 0.950, *P*_Egger’s_ = 0.869 and AA *vs.* GA/GG: *P* _Begg’s_= 0.703, *P*_Egger’s_ = 0.897 (Supplementary Figure S2).

## Discussion

Recently, variants in LEPR gene and their potential associations with cancer risk have been explored. Rs1137101 G>A polymorphism is one of the important variants in *LEPR* gene. *LEPR* rs1137101 G>A polymorphism is located on the exon region of LEPR gene, and it has been thought to be involved in the development of cancer by a number of studies. Several case–control studies reported that *LEPR* rs1137101 G>A polymorphism might be associated with the decreased risk of cancer [16,22,26,41,42]. However, several primary studies also suggested that *LEPR* rs1137101 locus could promote the progression of cancers [15,18]. Meanwhile, two meta-analyses were carried out to clarify the correlation of this SNP with susceptibility of overall cancer [9,10]. The results of these meta-analyses indicated that *LEPR* rs1137101 G>A polymorphism might not be associated with the risk of cancer. Moreover, more epidemiologic data were reported [11–26]. Therefore, an updated meta-analysis is necessary to calrify this issue precisely. In this meta-analysis, data of 44 case–control studies involving 13,711 cases and 22,225 controls, which is higher compared with these previous pooled-analyses mentioned above, were included and analyzed. Therefore, the obtained results may be more convincing.

LEP/LEPR signaling may promote cell proliferation and inhibit epithelial cell apoptosis [6]. In addition, Ben et al. [49] reported that *LEPR* rs1137101 G>A SNP may affect plasma LEP levels and BMI. A previous study suggested that leptin level was associated with the development of breast cancer [50]. However, null associations of *LEPR* rs1137101 locus with cancer susceptibility was identified, which was analogous to the results reported in previous meta-analyses [9,10,51,52], but unlike the other four meta-analyses [48,53–55]. Due to lack of sufficient data, the findings of previous systematic reviews and meta-analyses might be conflicting. Ethnicity also may be a vital factor for the difference. The minor allele frequency of *LEPR* rs1137101 G>A polymorphism was difference among different populations, but in stratified analysis by race, null relationship was found. Additionally, results of stratified analyses by sample size and source of control both found no relationship between *LEPR* rs1137101 G>A polymorphism with overall cancer susceptibility, highlighting that these variables could not influence the negative findings either.

According to the findings of stratified analysis, we found that *LEPR* rs1137101 locus might be associated with the susceptibility of oral and oropharyngeal cancer. However, the increased risk of cancer was dubious and hard to explain. Sample size was an important factor to determine the relationship between *LEPR* rs1137101 G>A polymorphism and cancer risk. In this subgroup, only 460 oral and oropharyngeal cancer cases and 503 controls were included for analysis, the findings might be underpowered.

Significant heterogeneity was found among the eligible studies in multiple genetic models. Thus, we carried out stratified analysis by ethnicity, cancer type, sample size, and source of control. It was obvious that Caucasians, hepatocellular carcinoma, breast cancer, other cancers, small sample sizes (<1000 subjects) and hospital-based case–control studies might contribute to heterogeneity.

In this meta-analysis, as mentioned in results, no publication bias was detected. In addition, findings of sensitivity analysis also indicated that our observation was convincing. Overall, results of this meta-analysis were stable and credible for the studied populations.

However, there were several limitations in the present pooled-analysis. First, some small sample size studies were recruited in this meta-analysis, which could promote the power of study. On the other hand, they led to potential publication bias and significant heterogeneity as well. Second, our findings were based on the crude pooled-results of the eligible case–control studies, the adjustment on characteristics and risk factors (e.g. BMI, physical exercise, age, gender, smoking, alcohol consumption, vegetable and fruit intake, and so on) was not performed. In the future, a more detailed assessment is needed, in which the characteristics and potential risk factors should be considered to adjust the findings. Third, although the cases and controls in eligible studies were fully matched, there was heterogeneity in different cancer types which may influence our results. Fourth, only PubMed and Embase databases were searched to retrieve the eligible. Finally, significant heterogeneity was found in all genetic models, which might influence the findings of our study. Thus, these results should be interpreted with caution.

In summary, this meta-analysis may be the largest sample size so far to assess the potential association of cancer risk with rs1137101 G>A polymorphism in *LEPR* gene. There is no significant association of cancer risk was identified to be correlated with rs1137101 G>A variants in the overall comparison, and the similar findings was also found in stratified analysis by ethnicity, cancer type, sample size, and source of control. In the future, more large-scale studies are needed to confirm or refute our findings.

## Supporting information

**Supplementary Figure S1:** 

**Supplementary Figure S2:** 

## Abbreviations

BMI: body mass index
CI: confidence interval
HWE: Hardy–Weinberg equilibrium
LEP: leptin
LEPR: leptin receptor
OR: odds ratio
SNP: single nucleotide polymorphism
