# Supplementary material for: Investigation of leptin receptor rs1137101 G>A polymorphism with cancer risk: evidence from 35936 subjects
Source: Biosci Rep. 2019 Jun 28;39(6):BSR20182240. doi: 10.1042/BSR20182240 (PMC6597850; doi:10.1042/BSR20182240)
Supplement: Supplementary file 1 [file bsr20182240_Supp1.pdf]

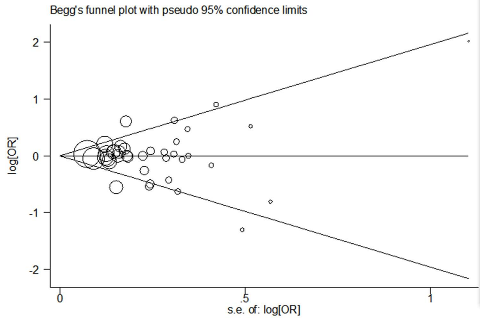

Begg's funnel plot with pseudo 95% confidence limits
log[OR]
2
1
0
-1
-2
0
.5
1
s.e. of: log[OR]

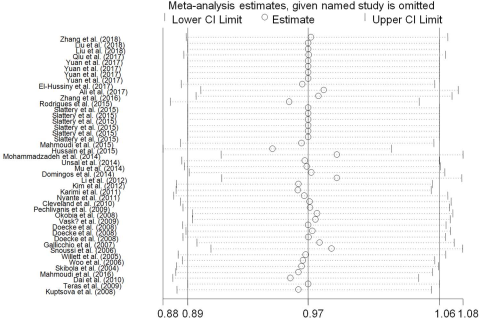
Meta-analysis estimates, given named study is omitted
Lower CI Limit
Estimate
Upper CI Limit
Zhang et al. (2018)
Liu et al. (2018)
Liu et al. (2018)
Qiu et al. (2017)
Yuan et al. (2017)
Yuan et al. (2017)
Yuan et al. (2017)
Yuan et al. (2017)
El-Hussiny et al. (2017)
Ali et al. (2017)
Zhang et al. (2016)
Rodrigues et al. (2015)
Slattery et al. (2015)
Slattery et al. (2015)
Slattery et al. (2015)
Slattery et al. (2015)
Slattery et al. (2015)
Slattery et al. (2015)
Mahmoudi et al. (2015)
Hussain et al. (2015)
Mohammadzadeh et al. (2014)
Unsal et al. (2014)
Mu et al. (2014)
Domingos et al. (2014)
Li et al. (2012)
Kim et al. (2012)
Karimi et al. (2011)
Nyante et al. (2011)
Cleveland et al. (2010)
Pechlivanis et al. (2009)
Okobia et al. (2008)
Vask? et al. (2009)
Doecke et al. (2008)
Doecke et al. (2008)
Doecke et al. (2008)
Gallicchio et al. (2007)
Snoussi et al. (2006)
Willett et al. (2005)
Woo et al. (2006)
Skibola et al. (2004)
Mahmoudi et al. (2016)
Dai et al. (2010)
Teras et al. (2009)
Kuptsova et al. (2008)
0.88
0.89
0.97
1.06
1.08
